# Unsupervised Medication-Induced Abortions: A Cross-Sectional Study in a Tertiary-Care Hospital

**DOI:** 10.7759/cureus.83049

**Published:** 2025-04-26

**Authors:** Preetam K Lenka, Manoj Kumar Mohanty, Saubhagya K Jena, Sudipta Singh, Jyotiranjan Sahoo

**Affiliations:** 1 Forensic Medicine, Institute of Medical Sciences (IMS) and Sum Hospital, Siksha 'O' Anusandhan (SOA), Bhubaneswar, IND; 2 Forensic Medicine, All India Institute of Medical Sciences, Bhubaneswar, Bhubaneswar, IND; 3 Obstetrics and Gyenacology, All India Institute of Medical Sciences, Bhubaneswar, Bhubaneswar, IND; 4 Community Medicine, IMS and Sum Hospital, Siksha 'O' Anusandhan, Bhubaneswar, IND

**Keywords:** induced abortion, medical abortion, medical abortion pills, mtp act, unsafe abortion, unsupervised medication

## Abstract

Background

Abortion refers to the termination of pregnancy before fetal viability, which is legally recognized at 28 weeks in India. Unsafe abortions, characterized by the absence of medical supervision, pose significant risks, including incomplete abortion, retained products of conception, and elevated maternal morbidity and mortality. Despite the Medical Termination of Pregnancy (MTP) Act (1971) and the introduction of Medical Abortion Pills (MAP), unsafe abortions remain prevalent.

Objective

To determine the prevalence of induced abortions through unsupervised use of MAP and to identify socio-demographic and obstetric factors associated with these cases, reported to the All India Institute of Medical Sciences (AIIMS), Bhubaneswar.

Methods

This hospital-based cross-sectional study enrolled 81 female patients aged 14-49 years, presenting with complications of unsupervised medical abortions. Data were collected using Case Report Forms (CRFs) and analyzed using SPSS v20. Statistical associations were assessed with chi-square or Fisher’s exact tests.

Results

The prevalence of unsupervised medical abortions was 67 out of 81 (82.7%). Age of the subject was weakly associated with a number of abortions, but statistically significant (r = 0.238, p = 0.020). Socioeconomic status (χ^2^ = 12.96, df = 3, p = 0.011) and gravidity [Odds Ratio (OR) = 4.5, p=0.014] showed significant associations. 62 (76.5%) patients were multi-gravida, and incomplete abortions were found in 53 (79.1%) patients. The association of a number of offspring with self-inducement of medical abortion was weak but statistically significant (r = 0.336, p = 0.001). The number of years married were weakly correlated with the number of abortions, and statistically significant (r = 0.314, p = 0.003). Statistically significant higher incidence of pregnancy was confirmed by ultrasonography (χ^2^ = 6.94, df = 1, p <0.001). In 40 cases (59.7 %), husbands had brought MAP, and in 48 patients (71.6%), consent was taken from their husbands for medical abortion. Pearson chi-square value for association between outcome of MAP and gestational age for induction of abortion was 16.049, which was found to be statistically significant (p = 0.025). Mean days of gestation of the self-medicated group and facility-induced group were 44.76 ± 32.2 and 119.5 ± 21.7 days, respectively, for which association was found to be significant (χ^2^ = 36.7, df = 1, p < 0.001). Facility-induced abortions were primarily therapeutic (8 out of 14, 57.1%). Bleeding per vaginum was the most common presenting complaint, followed by abdominal pain.

Conclusion

Unsupervised use of MAP constitutes a significant proportion of induced abortions, driven by socioeconomic factors and limited access to regulated care. Incomplete abortions remain a critical issue. Strengthening regulatory measures, promoting awareness, and improving access to safe abortion services are imperative for reducing maternal morbidity and achieving sustainable health outcomes.

## Introduction

The term “Abortion” is derived from the Latin word “*abortio*,” meaning to detach from a proper site [1]. Abortion is defined as the termination of pregnancy before fetal viability, which is accepted at 28 weeks in India [2]. Legally, terms like “abortion,” “miscarriage,” and “premature labor” are used synonymously to signify pregnancy termination before full term. Medically, expulsion within the first three months is referred to as abortion, miscarriage is for the gestation period between four to seven months, and beyond seven months but before term is designated as premature labor [3].

Abortions can be classified as natural (spontaneous or accidental) and artificial or induced [4]. Induced abortions, depending on the presence or absence of medical supervision, are categorized as therapeutic or criminal. Therapeutic abortions, also called justifiable or legal or safe abortions, are performed by registered medical practitioners (RMPs) under legal provisions to save or improve the mother’s health. In contrast, criminal abortions or illegal abortions, or unsafe abortions, occur without medical oversight [5]. The Declaration of Oslo attracted global think tankers to address the issues related to abortion from a legal perspective. WHO considers unsafe abortion as the termination of pregnancy by an unauthorized person at an inappropriate place that does not fulfill the required basic medical standards [6]. Usually, an act of criminal abortion is not detected unless it becomes unsuccessful [7]. It results in a significant increase in the risks of incomplete abortion and complications such as damage to the genito-urinal tract or retained products of conception (RPOC), leading to bleeding per vaginum and pain abdomen, etc., which in turn compromise maternal health [8]. So, unsafe abortions are associated with higher maternal morbidity and mortality, prompting governments worldwide to implement strict regulations.

In India, the Medical Termination of Pregnancy (MTP) Act of 1971 and its subsequent amendments, including the latest one of 2021, have been formulated to prevent unsafe abortions. It exclusively authorizes RMPs to prescribe medical abortion Pills (MAP) and ensures that facilities must be government-approved with surgical backup to deal with probable complications [9]. By means of medical abortion, the Drug Controller General of India (DCGI) approved Mifepristone in 2002 and Misoprostol in 2006 for terminating pregnancies up to 49 days. A combination pack of Mifepristone (1 tablet of 200 mg) and Misoprostol (4 tablets of 200 mcg each) was approved for use up to 63 days [10].

Social stigma surrounding unintended pregnancies, adolescent pregnancies, and unmarried pregnancies limits accurate data collection, with official statistics likely underreporting the true extent of the issue. Out of 42 million worldwide annual induced abortions, 19 million are unsafe abortions, causing approximately 13 % of all maternal deaths. In India, out of 6.4 million annual abortions, 56% are unsafe, accounting for 8-20% of maternal deaths [11]. Despite over 50 years of MTP Act enforcement and its subsequent multiple amendments, unsafe abortions remain widespread. Out of total induced abortions, around 81% are medication abortions, and that too mostly (91 %) outside the designated facility-based compared to only 7% in private facilities and 2% in public facilities, respectively [12]. MAP is often sold over the counter without prescriptions, and self-medication is increasing. This unsupervised use leads to adverse physical, mental, and social consequences. In the last decade, although India’s maternal mortality ratio (MMR) has improved from 174 in 2015 to 97 in 2020, as compared to the global status of 211 but the Sustainable Development Goal (SDG) target of 70 per 1,00,000 live births by 2030 is yet to be achieved [13]. Addressing unsafe abortion practices is critical for achieving this goal and improving women's reproductive health outcomes. The present study was carried out to quantify the cases of induced abortions following the intake of MAP without consulting any registered medical practitioner. It aims to address gaps in reproductive healthcare, focusing on factors like socioeconomic status, educational levels, and access to care. Findings reveal barriers such as inadequate healthcare infrastructure, social stigma, and ignorance about legal abortion provisions, which in turn delay appropriate care and increase complications.

## Materials and methods

The study was started after approval from the Institute Ethics Committee, All India Institute of Medical Sciences (AIIMS), Bhubaneswar (IEC/AIIMS/PG Thesis/2017-18/11) dated May 8, 2017. Written informed consent was obtained from study participants prior to enrollment in the study.

Objectives

The primary objective of the study was to determine the prevalence of induced abortions through unsupervised use of MAP among cases of induced abortion reported to AIIMS, Bhubaneswar. The secondary objectives were (a) identifying the association of various socio-demographic and obstetric factors with induced abortion, (b) examining the patterns of presenting complaints in cases of induced abortion through unsupervised use of MAP, and (c) understanding the pathway to care in such cases of induced abortion by unsupervised use of MAP.

Data collection

The study design was a hospital-based cross-sectional study during the study period from May 2017 to August 2018 comprised all consecutive cases of female patients of reproductive age, i.e., who met the inclusion and exclusion criteria. (Table 1) The study tool consisted of a Case Report Form (CRF) that captured detailed information on the followings, such as Demographic Indicators: Residential status, age, religion, marital status, family size, educational status, and occupational status and Medical Indicators: Menstrual history, obstetric history (pregnancy status, abortion history), and treatment history of the current condition.

**Table 1:** Study criteria

Inclusion Criteria	Female patients aged 14 to 49 years who provided informed consent.
	Patients presenting with complications from induced abortion due to unsupervised self-medication with MAP in the most recent pregnancy.
	Patients seeking therapeutic or legalized abortions under the supervision of a registered medical practitioner as per the MTP Act.
Exclusion Criteria	Patients diagnosed with a spontaneous abortion.
	Patients diagnosed with criminal abortion using methods other than MAP.
	Severely ill patients unable to participate in the study.

Patients who met the inclusion and exclusion criteria were recruited after being provided verbal and written information in a comprehensible language, supported by a Participant Information Sheet (PIS). Written consent was obtained using a Participant Informed Consent Form (PICF). All data were self-reported by patients, introducing a potential for recall bias.

Data confidentiality was maintained by using unique study codes instead of identifiable patient information. Data were entered in an Excel spreadsheet and analyzed using SPSS version 20 (IBM, Chicago). Categorical variables were expressed as numbers and percentages, with associations analyzed using the chi-square test. Quantitative variables were reported as mean and standard deviation (SD). A p-value of <0.05 was considered statistically significant.

## Results

During the study period, a total of 81 participants were enrolled. Out of which, 67 reported to be cases of unsupervised self-medication for abortion, and the rest were facility-induced abortion cases. Prevalence of induced abortion by means of unsupervised self-medication of MAP among the induced abortions was 82.7%.

Prevalence = No. of cases of unsupervised self-medication for abortion *100 /No. of cases of unsupervised self-medication for abortion + facility-induced abortions at AIIMS, Bhubaneswar

Around two-thirds of the study population were in the age group of < 30 years. The mean age of the study population was 28 years, 27.9 ± 5.3 years in the self-medicated group, whereas 29.4 ± 5.3 years in the facility-induced group. The data regarding age-groups <30 years and >30 years were represented in a 2 × 2 contingency table, and Fisher’s exact value was calculated, which was found not to be significant (p = 0.525). The distribution of the study population across urban and rural settlements was comparable to each other. Fischer’s exact value was calculated, which was not significant (p = 0.772). There was no statistically significant difference between the religions for induced abortion (p = 0.581) (Table 2).

**Table 2:** Association of sociodemographic factors with induced abortion.

Characteristics		Unsupervised self-medication (MAP)		Facility-induced (MTP)		Total		P-value
		n	%	n	%	n	%	
Age	< 30 years	49	73.1	9	64.3	58	75.6	0.525
	> 30 years	18	26.9	5	35.7	23	28.4	
Residential status	Urban	33	49.3	6	42.9	39	48.1	0.663
	Rural	34	50.7	8	57.1	42	51.9	
Religion	Hindu	62	92.5	14	100	76	93.8	0.581
	Muslim	5	7.5	0	0	5	6.2	
Marital Status	Married	63	94	14	100	77	9.1	0.597
	Unmarried	4	6	0	0	4	4.9	
Years of marriage	<2 years	8	12.7	5	35.7	13	16.9	0.140
	>2 years	55	87.3	9	64.3	64	83.1	
Education	Up to high school	25	37.3	3	21.4	28	34.6	0.359
	Beyond high school	42	62.7	11	78.6	53	65.4	
Occupation	Not gainfully employed	54	80.6	10	71.4	64	79	0.478
	Gainfully employed	13	19.4	4	28.6	17	21	
Socioeconomic status	Lower and Lower-middle class	1	1.5	3	21.4	4	4.9	0.011
	Middle class	12	17.9	1	7.2	13	16.1	
	Upper-middle class	22	32.8	7	50	29	35.8	
	Upper class	32	47.8	3	21.4	35	43.2	

Sixty-three subjects from the self-induced medical abortion group were married, in contrast to all patients in the facility-induced abortion group, showing no significant association (p = 0.597). The majority of patients were married for ≥ 2 years. Fischer’s exact value was calculated, which was not significant (p = 0.140). The number of years married were weakly correlated with the number of abortions, and the finding was statistically significant (r = 0.314, p = 0.003).

The mean years of schooling was 11.8 ± 3.08 years. Fischer’s exact test was applied to find the association of mean years of schooling with the self-inducement of medical abortion, which was not found to be statistically significant (p = 0.359). There was no significant association between education and the idea of abortion (p = 0.166). Gainfully employed refers to someone who is working in a job that provides regular income and is considered productive or economically beneficial. The majority of the population were dependents, and only a small fraction was gainfully employed. Fischer’s exact test was applied to find the association of occupation with the self-inducement of medical abortion, which was not found to be statistically significant (p = 0.478). According to the modified B.G. Prasad Socioeconomic Classification (2017), the majority of the patient group belonged to the upper class. The association of socioeconomic status with self-induction of abortion is significant (p = 0.011).

The association of the number of offspring with self-inducement of medical abortion was weak but statistically significant (r = 0.336, p=0.001). There was no significant difference in the gender of previous offspring on the present abortion (p=0.698). The odds of self-medicated abortion among multi-gravida were higher (Odds ratio = 4.5, p = 0.014, 95% CI: 1.35 to 15.51). Pregnant status was perceived by patients either by delayed menstrual cycle (7.4%) or urine pregnancy test (53.5%), or both (39.2%). There was a statistically significantly higher incidence of pregnancy confirmation by USG (p <0.001). There was no association of history of previous abortions with self-medication for abortion (p = 0.228). There was a significant difference between the number of previous abortions in the self-medication and facility-induced groups (p = 0.027). Age of the subject was weakly associated with a number of abortions, but the finding was statistically significant (r = 0.238, p = 0.020). The mean days of gestation of the self-medicated group and facility-induced group were 44.76 ± 32.2 and 119.5 ± 21.7 days, respectively. Association was found to be significant between the two groups (p < 0.001). Evaluating the association, the Pearson chi-square value was 16.049, which was found to be statistically significant (p = 0.025) (Table 3).

**Table 3:** Association of obstetric characteristics with induced abortion

Characteristics		Unsupervised self-medicated (MAP)		Facility-induced (MTP)		Total		P-value
		N	%	n	%	n	%	
No. of off-springs	0	13	19.4	10	71.4	23	28.4	0.001
	1	28	41.8	2	14.3	30	37.0	
	>1	26	38.8	2	14.3	28	34.6	
Gender (n=58)	Only male	17	31.5	2	50	19	32.8	0.698
	Only female	24	44.4	1	25	25	43.1	
	Both	13	24.1	1	25	14	24.1	
Gravidity	Primigravida	12	17.9	7	50	19	27.5	0.014
	Multigravida	55	82.1	7	50	62	76.5	
Self-diagnosis of pregnancy	By menstrual cycle	5	7.5	1	7.1	6	7.4	0.944
	Urine pregnancy test	35	52.2	8	57.1	43	53.1	
	Both	27	40.3	5	35.7	32	39.5	
Pregnancy confirmation by USG	Yes	37	55.2	14	100	51	62.9	<0.001
	No	30	44.8	0	0	30	37.1	
History of abortion	Yes	28	41.8	3	21.4	31	38.3	0.228
	No	39	58.2	11	78.6	50	61.7	
No. of abortion	0	39	58.2	11	78.6	50	61.7	0.027
	1	26	38.8	1	7.1	27	33.3	
	>1	2	3.0	2	14.3	4	4.9	
Gestational age at induction of abortion	<30 days	14	20.9	0	0	14	17.3	<0.001
	30-63 days	46	68.7	0	0	46	56.8	
	>63 days	7	10.4	14	100	21	25.9	

Patterns of presenting complaints

**Figure 1:**
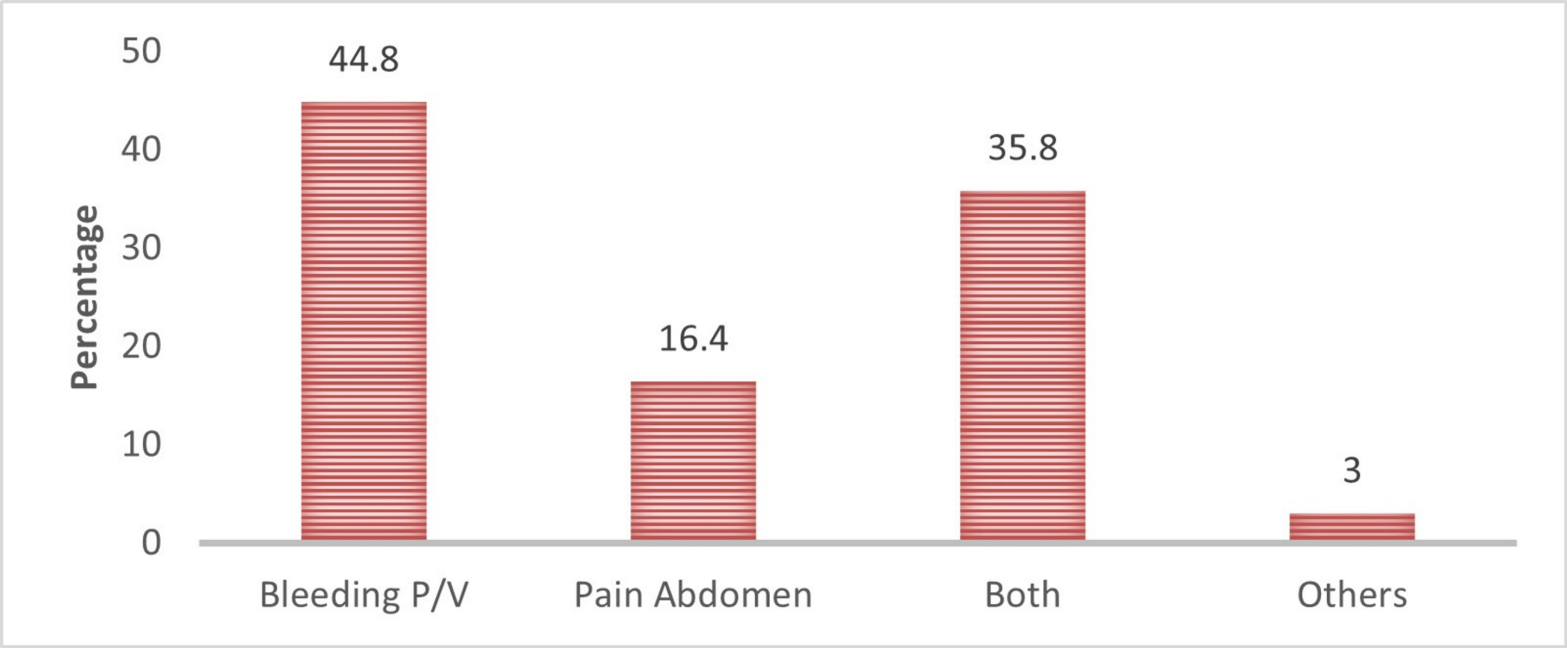
Patterns of presenting complaints

Bleeding per vaginum was the most common presenting complaint in patients taking self-medication for abortion, followed by abdominal pain (Figure 1).

Health-seeking behavior in cases of induced abortion

Self-medicated subjects (n = 67) had incomplete abortion in 53 patients (79.1%), complete abortion in nine patients (13.4%), and failed abortion in five patients (7.5%). In case of facility-induced safe abortion (n = 14), most of the MTPs were performed based upon therapeutic indication (n = 8, 57.1%), and the rest were eugenic (n = 6, 42.9%). It was not advised in a relatively large (n = 45, 67.2%) proportion of the population and was positively correlated with gestational age at which MAP was taken (r = 0.353, p = 0.005). 56 (84%) subjects in the self-induced abortion group already had a previous idea about abortion. In the majority of patients, the husband (n = 23, 34.3%) was the source of the idea, followed by a friend (n = 21, 31.3%) and the media (n = 8, 11.9%). A large proportion of patients (n = 48, 71.6%) took consent from their husbands to take medication for abortion. In most of the cases (n = 40, 59.7%), the MAP was brought by the husband, while in 17 (25.4%) patients by herself. Almost all the patients took medicine once, except one who took a second dose when the urine pregnancy test was still positive after the first dose. 20 subjects (29.9%) did not comply with the full dosage protocol for abortion. As AIIMS, Bhubaneswar is a tertiary-care center, patient consulted other healthcare facility or system easily accessible to them. 47 patients (70.1%) consulted RMP in other healthcare facilities, and five patients (7%) visited a system of alternative medicine before seeking consultation at our center. The mean number of days taken to visit our center after taking MAP was 17.31 ± 24.36 days, the median was seven days, and the interquartile range (IQR) was 26 days.

## Discussion

Though the study population was homogeneous, the generalizability of results may be limited due to referral bias.

Peak age group of < 30 years of the study population of unsupervised self-medication of MAP for abortion is comparable to findings of studies by Munshi et al., Armo et al., Agrawal and Datta, Giri et al., and Thaker et al. [10,11,14-16]. The mean age of participants (27 ± 5.3 years) aligns with studies by Singh et al. [17]. High fertility rate and high mating frequency may be the reasons for the corresponding peak age group. AIIMS, Bhubaneswar, being a referral institute, cases not only came from different districts of the state but also from nearby states like West Bengal and even as far as Maharashtra. Most participants were Hindus and from rural areas, consistent with findings of a study by Thaker et al., likely due to the demographic composition of the study area [11].

Similar to Thaker et al. and Panda et al., most of the study population of induced abortion were married [16,18]. All facility-based cases were married women, which signifies unmarried women often chose unsafe abortions, potentially due to social stigma. The recent marriage was identified as ≤ 2 years at the time of abortion since marriage. Patra noted that recently married women were less likely to use unsupervised medication, aligning with our findings [19].

Education played a significant role, with two-thirds of participants having at least 10 years of schooling, echoing Armo et al. [11]. Educated women may detect pregnancies earlier but face barriers in accessing facility-based services, either public or even private set up; may be due to social constraints and fear of breach of secrecy. The majority of cases were from upper socioeconomic classes (modified BG Prasad scale, 2017), indicating a preference for better healthcare only after encountering complications at the cost of privacy, though recall bias could not be ruled out [20]. Only one-fifth were gainfully employed, suggesting financial independence influences decision-making and access to safer options.

Participants of induced abortion were mostly multigravida, consistent with Singh et al. and Panda et al.’s studies [17,18]. More than 80 % of the self-induced study population had at least one or more living offspring, similar to Munshi et al., who reported higher abortion rates with increased parity [10]. However, the different results of Agarwal et al. may be due to population and geographical variations [14].

Most participants self-diagnosed pregnancy using urine tests and menstrual cycles, with 55% confirming via ultrasonography, similar to findings of Thaker et al. [16]. This double-checking reflects a determined decision to terminate pregnancy. Bleeding per vaginum was the most common complaint, followed by abdominal pain, consistent with Singh et al., Thaker et al., and Sharma et al. [12,16,21]. About four-fifths of cases resulted in incomplete abortions, echoing with Nivedita et al. and Sarojini et al, necessitating surgical interventions in two-thirds of cases, similar to findings by Dasgupta et al. [22-24]. Five cases of failed abortions were reported to be caused by faulty dosing of MAP. Two cases of ectopic pregnancies could be the adverse outcome of the MAP, highlighting the need for ultrasonography before medical abortion, as suggested by Giri et al. and Thaker et al. [15,16].

Many women lacked autonomy in decision-making, influenced by education, occupation, and social constraints. Improper awareness of abortion laws, as noted by Singh et al., and barriers like limited access, confidentiality issues, and family support, as highlighted by Armo et al. and Thaker et al., deterred facility-based care [11,12,16]. Husbands were often the primary decision-makers and providers of MAP, though poor understanding of dosage and side effects, coupled with inadequate guidance from an inappropriate service provider, led to complications as mentioned by Agarwal et al. [14].

Unawareness of the MTP Act, 1971, and its latest 2021 amendment, as well as provisions of different sections of BNS (Bharatiya Nyaya Sanhimta), 2023, further complicates the situation. Consent from the woman is sufficient for termination, yet societal norms often demand spousal approval. Legal ambiguities, such as the 20-week gestation cutoff excluding seven special scenarios, and the inclusion of non-allopathic practitioners in abortion services, compromise quality and safety [25]. WHO’s criteria for quality care - safety, accessibility, and acceptability - are often unmet in public sector services due to limited resources and inadequate infrastructure.

Addressing these issues requires a multifaceted approach: enhancing women’s education and empowerment, improving infrastructure and workforce, raising public awareness of abortion laws, and ensuring access to quality maternal and reproductive healthcare to mitigate the physical, mental, and social impacts of unsafe abortions.

This study has a few limitations. Being a single-center study conducted in a tertiary-care hospital, the findings may not be generalizable to the broader community. The cross-sectional design restricts the ability to establish causality between associated factors and unsupervised medical abortion. The relatively small sample size may have limited the statistical power to detect weaker associations. Additionally, the reliance on self-reported data for sensitive issues such as abortion practices may introduce recall bias or social desirability bias. The study also did not evaluate long-term outcomes or complications following unsupervised abortions, nor did it capture detailed information on the type, source, or dosage of MAPs used.

## Conclusions

The study found a high prevalence (82.7%) of unsupervised medical abortion, with significant associations with socioeconomic status, gravidity, gestational age, and spousal involvement, while factors like age, years of marriage, and number of offspring showed weak but statistically significant correlations with abortion practices. Unsupervised self-medicated abortions are being reported at a catastrophic prevalence rate to healthcare facilities due to complications like heavy bleeding or abdominal pain. The legal acts and provisions are not enough to curtail the bundle of chaos created by this avoidable problem. Public awareness regarding the severity of complications and measures of circumvention can be created to achieve desirable outcomes for ensuring better maternal health.
